# Presumptive Treatment of Malaria in Ghana: Was It Ever Useful? Evidence from the Kassena-Nankana District of Northern Ghana

**DOI:** 10.1155/2018/3408089

**Published:** 2018-07-08

**Authors:** Michael N. K. Babayara, Bright Addo

**Affiliations:** ^1^Postgraduate College, 37 Military Hospital, Accra Central, Ghana; ^2^University of Ghana School of Public Health, Legon, Accra, Ghana

## Abstract

**Background:**

The WHO currently advocates parasitological confirmation of malaria before treatment is commenced. However, many arguments have emerged both for and against this new position. To contribute to the debate, this secondary data analysis was conducted to determine the likelihood of malaria parasitaemia in a child presenting with fever, vomiting, or cough in the Kassena-Nankana District.

**Methods:**

The dataset for this analysis was generated during a study to assess the incidence and risk factors for paediatric rotavirus diarrhoea in the Kassena-Nankana District. Over a two-year period, trained field staff recruited 2086 subjects with episodes of diarrhoea aged 24 months or below into the study. A standard case report form was used to collect data on histories of illness, symptoms, vaccination, and anthropometry. Blood smears were tested for malaria parasites. The data set generated was obtained, cleaned, and analysed using Epi Info version 7.1.1.14 statistical software.

**Results:**

Of the 2086 subjects recruited, 2078 had blood smears done and 54.0% had malaria parasites. Fever and vomiting appeared to be associated with parasitaemia with odds ratios of 1.9 (95% CI: 1.5586–2.2370) and 1.2 (95% CI: 1.0352–1.4697), respectively. Cough however appeared protective with an odds ratio of 0.8 (95% CI: 0.6910–0.9765). The odds of parasitaemia appeared to increase where a child presented with more than one symptom.

**Conclusion:**

Nearly half (46%) of the subjects in this study presented with symptoms but had no malaria. Presumptive treatment of malaria may therefore be useful in situations where diagnostic tests are not readily available, its routine practice should however not be encouraged.

## 1. Introduction

Malaria still remains one of the greatest threats to the survival of the Sub-Saharan African child today. In 2015, an estimated 212 million cases of malaria occurred globally, out of which the WHO African Region accounted for 90.0% of these cases, followed by the WHO South-East Asia Region (7.0%) and the WHO Eastern Mediterranean Region (2.0%) [1].

To ensure prompt treatment and avert the possibility of mortality, a practice of presumptive treatment was widely advocated by many. The WHO in its 2006 treatment guidelines advocated treatment of malaria on grounds of clinical suspicion in areas which have high stable transmission for children less than 5 years [2]. In accordance with this directive, malaria treatment in many parts of Africa and Asia where the disease burden is greatest was often dispensed on the basis of certain symptoms which were deemed malaria symptoms. These symptoms included fever, vomiting, anaemia, diarrhoea, cough, and fast breathing. The Integrated Management of Childhood Illnesses (IMCI) approach recommended clinicians and caregivers to put together these symptoms and signs and make a meaningful guess regarding the diagnosis of malaria [3].

Unfortunately, these symptoms singularly or in combination with other symptoms may be caused by other equally life threatening conditions such as urinary tract infections, meningitis, acute viral hepatitis, enteric fever, yellow fever, gastroenteritis, acute respiratory infections, pneumonia, and many more [4]. The real danger with presumptive treatment therefore is not just over diagnosis of malaria, but also misdiagnosis and subsequently missed opportunities to correctly treat a sick child.

The argument for or against the practice of presumptive treatment of malaria is one that is still raging on even though the WHO has since revised its position on the subject. The latest WHO Guidelines for the Treatment of Malaria state that “all cases of suspected malaria should have a parasitological test (microscopy or rapid diagnostic test) to confirm the diagnosis” [5]. This latest revision is based on a number of assumptions which have been contested by some scholars. The WHO assumes that clinical diagnosis has a low specificity and results in overtreatment whiles parasitological confirmation prevents unnecessary use of antimalarials, improves patient care, improves case detection and reporting, and gives room for considering alternative diagnoses [2].

This position of the WHO is shared by many. One significant argument advanced is that malaria transmission has over the years declined so much that the once so-called highly endemic areas have now become less endemic. For example, there is a sharp decline in Sub-Saharan Africa of the* Plasmodium falciparum* prevalence rate in children aged 2-10 years from 37.0% in the years 1985–1999 to 17.0% in 2007. In the face of evidence like this, fevers among children can no more be presumed to be from malaria. If this fact is ignored and malaria is still presumed to be the cause of every fever, then a lot of diagnoses will be missed and this will definitely have implications on morbidity and mortality [6, 7].

A difficult challenge to evidence-based treatment of malaria is how to obtain laboratory confirmation in deprived settings. However, the introduction of malaria rapid diagnostic tests (RDTs) provides an answer to this challenge. RDTs have been shown to be reliable, easy-to-use, and inexpensive diagnostic tests. RDTs have a good sensitivity, require minimal training and retain their accuracy even after extensive storage under tropical conditions. Two separate meta-analyses have demonstrated that the performance of RDTs is comparable to that of expert microscopy [8, 9].

In routine practice, these diagnostic tools have proved to be of high performance if only they are carried out well [10]. Indeed, some studies in Ghana have already concluded that RDT based management of malaria is likely to be acceptable to care givers and will integrate well into the IMCI context [11]. While these arguments may sound reasonable and convincing, counterarguments have been made by other schools of thought to discredit them.

In an attempt to contribute to the debate and verify whether the mere presence of the so-called “malaria symptoms” is enough to warrant an antimalarial prescription, this study was designed. It is a secondary data analysis in which we sought to determine whether the mere presence of symptoms deemed to be malaria symptoms is associated with an increased likelihood of malaria parasitaemia in a child in the Kassena-Nankana District (KND) of Northern Ghana; and if it is associated, is the association strong enough to warrant an antimalarial prescription without laboratory confirmation? We hypothesized that presenting with vomiting, fever, and/or cough is associated with an increased likelihood of malaria parasitaemia in infants in the KND.

## 2. Materials and Methods

### 2.1. Study Area

**Figure 1:**
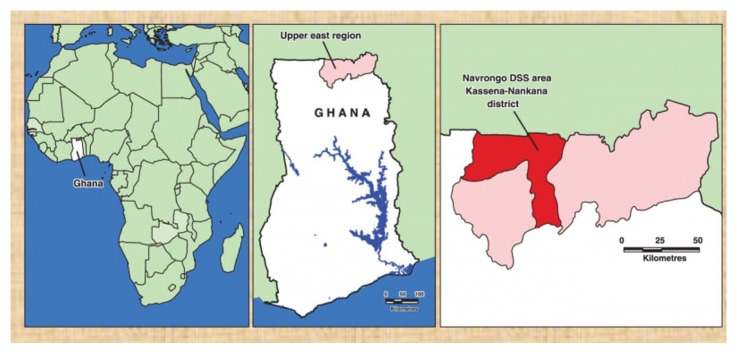
Map of Ghana showing the DSS area in the Kassena-Nankana District.

The dataset for this analysis was generated during a study to assess the “incidence and risk factors for paediatric rotavirus diarrhoea in northern Ghana”, a study carried out in the Kassena-Nankana District of the upper east region of Ghana. The KND is one of the 230 districts in Ghana and lies in the Guinea Savannah belt. It is at the extreme north of the country (Figure 1) and shares borders with Burkina Faso, the northern neighbors of Ghana. KND has an area of about 1675 km^2^ and a population of about 160,000 and is one of the hyperendemic areas of malaria transmission. Navrongo, the district capital is home to one district hospital, and the Navrongo Health Research Centre (NHRC); one of the renowned health research institutions in the country. The Navrongo Demographic Surveillance System (NDSS) is run by the NHRC and monitors population dynamics in the area [12]. Each compound is visited by an NDSS fieldworker once every 90 days to register events such as pregnancies, births, deaths, and migrations. The main occupation of the people is subsistence farming, predominantly of cereals and livestock. KND has a mean monthly temperature range of 20–45°C and rainfall averages 800–1000 mm per annum, occurring mainly between May and October.

### 2.2. Data Collection

#### 2.2.1. Diarrhoea Surveillance

The study was based at three health facilities: the Paga health centre, Kassena-Nankana East (KNE) health centre, and the War Memorial Hospital in Navrongo (the district hospital). Trained field staff working in these three health facilities screened and recruited subjects with diarrhoea who were brought to the health facilities by mothers and caretakers. Children were eligible for inclusion in the study if they were aged no more than 24 months and had passed three or more loose stools per day, on any of the previous 3 days. Once recruited, subjects were then treated for their diarrhoeal episodes and those who were very sick were referred to the district hospital for further management. Subjects were not followed up for a mortality endpoint. Fieldwork lasted from August 1998 to July 2000.

A standard case report form was used to collect information on histories of illness, vaccination, and anthropometry. Height was measured using a length board, and weight using an electronic weighing scale. To permit calculation of the Vesikari score as a measure of diarrhoea severity, information was collected on duration of diarrhoea, peak number of stools passed per day, duration of vomiting and peak frequency of vomiting per day, degree of fever, presence and severity of dehydration, and treatment.

#### 2.2.2. Laboratory Procedures

A stool sample was taken if possible; otherwise a rectal swab was performed. The samples were transported to Accra for virus identification at the Noguchi Memorial Institute for Medical research. Detection of rotavirus was done by the enzyme-linked immunosorbent assay (ELISA) technique. Blood smears were taken by laboratory staff during field visits and blood films prepared and analysed for malaria parasites at the NHRC laboratory. Where films were positive, parasite load was determined by (1) counting the number of parasites amongst 200 white blood cells (WBCs), (2) dividing the number obtained by 200, and (3) multiplying the ratio by the total number of WBCs obtained from the patients full blood count (where available), or 8000 (WHO standard WBC count per uL). Mathematically, the parasite count could be expressed as Count/uL = Number of parasites/200 x Total WBC count of patient (or standard WHO figure of 8000).

### 2.3. Data Management and Statistical Analysis

The forms were double-entered into a FoxPro database in Navrongo and data cleaning done before analysis. On obtaining the data, the dataset was converted into an excel file and analysed using Epi Info version 7.1.1.14 statistical software. Using this software, frequencies were calculated for demographic characteristics of participants, while bivariate analysis was conducted to obtain the odds of a child getting a positive smear for malaria if he/she presented with various symptoms. Symptoms were fever, vomiting, and cough. Diarrhoea was excluded since it had a 100.0% frequency. In the calculations, parasites were exposure variables, while symptoms were outcome variables. Odds ratios were calculated with associated 95% confidence intervals.

### 2.4. Ethics Considerations

The “incidence and risk factors of paediatric rotavirus diarrhoea in the Kassena-Nankana District of the upper east region of Ghana” project from which we extracted data for our analysis was approved by the ethics committees of the London School of Hygiene and Tropical Medicine, Ghana Health Service, and Navrongo Health Research Centre. Community entry in the form of a community durbar with chiefs and people present was done before start of the study. Prior to data collection, consent forms were given to parents/primary caretakers before subjects were enrolled. We sought permission from the investigators before extracting the dataset.

## 3. Results

### 3.1. Characteristics of Study Participants and Symptoms Distribution

**Figure 2:**
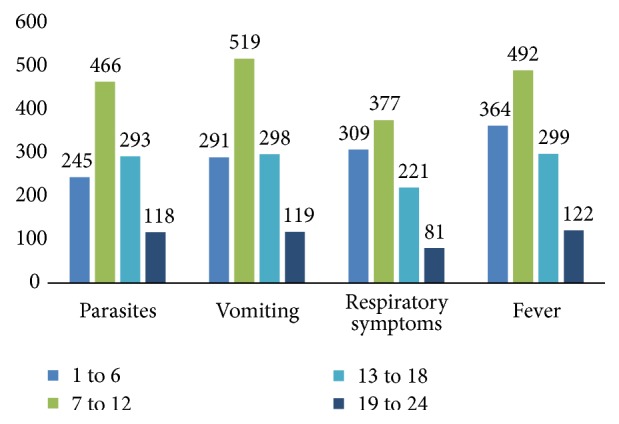
Distribution of parasites and symptoms by age groups (in months).

A total of 2086 children aged up to, but not above 2 years, were recruited into the study as follows. 1138 (55.0%) were males and 948 (45.0%) females. About 2078 of these children had blood smears performed out of which more than half the number of blood smears (54.0%) was positive. In terms of symptom distribution, diarrhoea was the baseline criterion for inclusion into the study and therefore had a 100.0% distribution. However, vomiting constituted the commonest symptom apart from diarrhoea, occurring in 58.8% of children compared to 47.4% cough, and 38.8% for fever (Figure 2 and Table 1). This distribution is perhaps not surprising considering the fact that vomiting is a key symptom of gastroenteritis alongside diarrhoea, the main inclusion criteria in this study.

Sex did not seem to influence the occurrence of parasites or symptoms in study subjects as 54.0% of males and females each carried parasites in their blood. Fifty-nine per cent (59.0%) of both sexes also presented with vomiting, whiles 48.0% for males and 46.0% for females presented with cough. Fever had the least prevalence of 37.2% and 40.7% for males and females, respectively. The distribution generally did not seem be influenced by age group either. However, infants between 7 and 12 months appeared to have higher prevalence rates of parasites and symptoms than all other age groups (Figure 2).

### 3.2. Seasonal Variation in Prevalence of Symptoms and Parasite Load (Dry versus Rainy Seasons)

**Figure 3:**
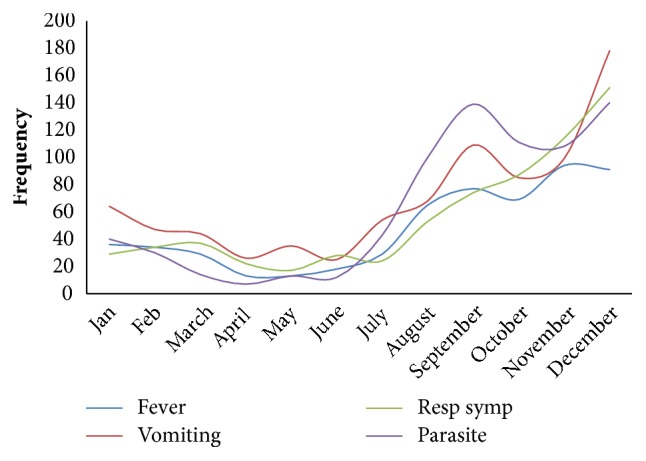
Distribution of symptoms and parasite load showing a rise during the Rainy Season (Jun to Nov).

The results showed a seasonal variation in the prevalence of symptoms and parasite load (Figure 3). Generally, more symptoms and parasite load were recorded during the rainy season (June to November).

### 3.3. Relationship between Symptoms and Malaria Parasitaemia

When individual symptoms were juxtaposed against parasites, fever appeared to be the symptom most associated with parasitaemia. The odds of testing positive for parasites given the presence of fever were 1.87(CI: 1.5586 - 2.2370). If children presented with vomiting, they had 1.23 Odds (CI: 1.0352-1.4697) of testing positive for parasites. Cough seemed to rather protect against the likelihood of parasites with an odds ratio of 0.82 (CI: 0.6910–0.9765). The odds of parasitaemia in a child increased as number of presenting symptoms correspondingly increased. Where a child presented with only one of the symptoms, the odds of parasites were 1.33. This increased to 1.36 for two symptoms and 1.88 for all three symptoms (Table 2).

## 4. Discussion

Malaria in Ghana accounted for about 35.0% of Out Patients Department (OPD) attendance in hospitals in the late 1990s and some researchers believe this prevalence has increased over the years. This figure, however, varies greatly from region to region depending on the sociodemographic characteristics of an area. The Kassena-Nankana District has over the last two decades been recognized as one of the most endemic regions for malaria in Ghana [13]. The parasite prevalence rate of 54.0% in this study corroborates this conclusion and gives further meaning to the push for the extensive use of insecticide treated mosquito nets in households. Indeed, an evaluation of the effectiveness of LLITNs in the area demonstrated a 17.0% drop in all case mortality rate in children aged 6 months to 4 years [13]. This achievement is in spite of the fact that there were difficulties with the mobilization of the mosquito nets. If these challenges are eventually addressed and the use of mosquito nets is successfully encouraged in the area, and other parts of the country, the fight against malaria will be partly won.

Parasite prevalence has never been shown to be influenced by sex in malaria transmission. This is further evident from the sex distribution of parasites in this analysis. Age, however, appears to influence this. Typically, neonates have been generally thought to be more immune to malaria than older children owing to the fact that maternal antibodies are usually present in them and confer some immunity. From age one month to one year however, babies become more susceptible and show high parasite rates as shown in this analysis. The decline in maternal antibodies when their own immune systems are not yet well developed may be responsible for this increased susceptibility.

The Kassena-Nankana District is generally characterized by a dry savanna climate. This situation however changes during the rainy season which extends from June to November. During this period, malaria prevalence increases as mosquito breeding places abound with pockets of water in various receptacles. It is therefore not surprising that this analysis shows higher parasite prevalence during the rainy season. Other studies in the study area have described similar trends [11, 14]. This rising trend in parasite rate is accompanied by a similar trend in the reported symptoms. This could be attributed to a general rise in sanitation related diseases (e.g., diarrhoeal disease) during this period as unsanitary conditions tend to abound during rainy seasons in many parts of Ghana. It could also be related to the observed rise in parasite prevalence.

Typically, malaria is characterized by bouts of fever, chills, and rigors. Other symptoms such as vomiting, jaundice, dyspnoea, and many others are usually present. Fever has been found to be one of the most common presenting symptoms and has therefore often led to presumptive treatment. Results in this analysis however showed parasitaemia in only 23.0% of children presenting with fever. This compares well with results in other studies which report 15.0% of fever cases with parasitaemia [15]. In this analysis, odds of parasitaemia were 1.87 where a child presented with fever. This looks significant; however, an argument that this is enough to conclude that fever alone can necessarily be equated to malaria is still not supported. Among the cohort of children with fever, the 77.0% who had no parasitaemia needed to have the cause of their fever investigated and treated. Laboratory confirmation in this case would have precisely distinguished the real malaria related fevers from the other fevers.

We found vomiting of children to be less indicative of malaria. This could be due to the fact that diarrhoea which was the main inclusion criterion in the recruitment of cases into this study often occurs together with vomiting as symptoms of gastroenteritis. Any association that may have existed between vomiting and parasitaemia will therefore be weak. It could also be a true indication that vomiting alone is not predictive of malaria as it is widely believed. In either case, the significance of this analysis remains the same; vomiting alone in general is not enough to indicate a presumptive diagnosis of malaria, and confirmatory laboratory tests would have been essential.

Cough generally is more of a symptom of respiratory disease than malaria even though complications of malaria may occasionally present with cough and rapid breathing. In this analysis, the apparently protective nature of cough against parasitaemia is an indication that a cough in a child is not likely to be due to malaria. Such a child should be carefully examined for other causes of coughs such as respiratory tract infections.

An increasing likelihood of parasitaemia when the number of presenting symptoms increases as is shown in our analysis is in keeping with the general rule in making clinical diagnosis in medical practice. Generally, the more a patient presents with signs and symptoms of a condition, the more likely it is that they have that condition. Indeed, it even becomes more certain where pathognomonic signs and symptoms (a particular sign/symptom whose presence means that a particular disease is present beyond any doubt) are present. In malaria however, no pathognomonic signs and symptoms exist, and the very classical signs and symptoms that suggest malaria also suggest other conditions such as enteric fever, urinary tract infections (UTI), respiratory tract infections, etc. A laboratory confirmation is therefore always necessary. The WHO's earlier call for presumptive treatment of malaria was in good taste and was intended to encourage prompt treatment and prevent unnecessary malaria deaths. However, considering the weight of the current evidence, it can only be hoped that many more deaths have not been caused instead.

## 5. Limitations

A primary limitation of our analysis is the use of a preexisting dataset which did not permit us to examine other symptoms beyond what was captured in the dataset, i.e., fever, diarrhoea, vomiting, and cough.

Added to this limitation was the limitedness in the age category of study subjects. Given the opportunity, we would have expanded the age bracket to include older children and stratified the analysis to determine if variations existed between age groups.

## 6. Conclusion

The results of our analysis showed that there was an association between malaria parasitaemia and symptoms such as fever, vomiting, and cough. The likelihood of parasitaemia also increased where these symptoms occurred together. However, not all children presenting with these symptoms had malaria parasitaemia. The implication of this finding is that, while it may be expedient to presumptively treat children suspected to be suffering from malaria in emergency situations and in instances where laboratory confirmation is not readily available, the routine use of this approach should not be encouraged. In these situations, presumptive treatment can be ongoing, while alternative diagnoses are actively considered to confirm or refute the presumptive diagnosis of malaria. This is necessary to prevent the over diagnosis of malaria and the misuse of antimalarials. Most importantly, this will help eliminate the risk of missing out on other potentially fatal diseases.

## Figures and Tables

**Table 1:** Distribution of parasites and symptoms by sex in study subjects (N=2086).

**Variable**	**Male (n=1138)**		**Female (n=948)**	
	**Yes**	**No**	**Yes**	**No**
**Parasites**	614(54.0%)	521(46.0%)	508(54.0%)	435(46.0%)
**Vomiting**	671(59.0%)	467(41.0%)	556(59.0%)	392(41.0%)
**Fever**	423(37.2%)	715(63.8%)	386(40.7%)	562(59.3%)
**Cough (Resp. symptoms)**	546(48.0%)	592(52.0%)	442(46.6%)	506(53.4%)

**Table 2:** Symptoms and Malaria Parasitaemia.

**Symptom **	**Odds Ratio (OR)**	**95% Confidence Interval**
Fever	1.8672*∗*	1.5586 – 2.2370
Cough (Resp. symptoms)	0.8214*∗*	0.6910 – 0.9765
Vomiting	1.2335*∗*	1.0352 – 1.4697
**Number of symptoms presented**		
None	1	
One	1.331*∗*	1.031 – 1.718
Two	1.359*∗*	1.050 – 1.761
Three	1.883*∗*	1.372 – 2.583

*∗p < 0.05*.

## Data Availability

Data for this analysis was obtained from the study conducted to assess the incidence and risk factors for paediatric rotavirus diarrhoea in northern Ghana.
